# Phase 1 study of ganitumab (AMG 479), a fully human monoclonal antibody against the insulin-like growth factor receptor type I (IGF1R), in Japanese patients with advanced solid tumors

**DOI:** 10.1007/s00280-012-1924-9

**Published:** 2012-07-19

**Authors:** Haruyasu Murakami, Toshihiko Doi, Nobuyuki Yamamoto, Junichiro Watanabe, Narikazu Boku, Nozomu Fuse, Takayuki Yoshino, Atsushi Ohtsu, Satoru Otani, Kazuhiro Shibayama, Takatoshi Takubo, Elwyn Loh

**Affiliations:** 1 Shizuoka Cancer Center 1007 Shimonagakubo, Nagaizumi-cho Sunto-gun, Shizuoka 411-8777 Japan; 2 National Cancer Center Hospital East, Kashiwa, Japan; 3 St. Marianna University School of Medicine Hospital, Kawasaki, Japan; 4 Takeda Bio Development Center Ltd., Tokyo, Japan; 5 Amgen Inc., San Francisco, CA USA

**Keywords:** Clinical trial, phase 1, Ganitumab, Gemcitabine, Insulin-like growth factor receptor type 1

## Abstract

**Purpose:**

This study was to investigate the safety and tolerability of ganitumab in Japanese patients with advanced solid tumors.

**Methods:**

Patients were enrolled into 1 of 3 dose cohorts (6, 12, or 20 mg/kg) of single-agent ganitumab administered intravenously every 2 weeks. Dose-limiting toxicity (DLT) was assessed for the first 28 days. The primary objectives were to assess the safety, tolerability, and pharmacokinetics (PK) of ganitumab in Japanese patients with advanced solid tumors. An exploratory pharmacodynamic analysis was done to investigate the relationship between exposure and changes in the level of circulating factors in IGF1R pathway (IGFBP-3 and total IGF-1).

**Results:**

Nineteen patients with ECOG performance status 0–1 (6 in cohort 1 and 3, 7 in cohort 2) received at least 1 dose of ganitumab. Median age was 58.0 years. Tumor types included: breast (4), gastric (3), rectal (2), NSCLC (2), thymic (2), and other cancers (6). No DLTs were observed. The most common grade ≥3 adverse events were neutropenia (21 %), leukopenia (16 %) and lymphopenia (11 %). There was a trend of dose-dependency on severity of thrombocytopenia, but not on that of neutropenia. No neutralizing anti-ganitumab antibodies were detected during this study. Dose-linearity on PK of ganitumab was indicated in the dose range. Tumor response was assessed for 19 patients. Stable disease as best response was reported in 7 patients.

**Conclusions:**

Ganitumab up to 20 mg/kg was tolerable in Japanese patients with advanced solid tumors. The safety and PK profiles were similar to those previously observed in non-Japanese patients.

## Introduction

The insulin-like growth factor receptor type 1 (IGF1R), a transmembrane receptor of tyrosine kinase [1], is expressed ubiquitously in the normal adult, except hepatocytes and mature B cells. This receptor is an integral component of the physiologic mechanism controlling organ size and homeostasis [2]. Most human cancer cells also express IGF1R [3–5].

Experimental evidence indicates that IGF1R signaling supports tumor growth and survival predominantly through stimulation of the phosphoinositide 3-kinase (PI_3_K), an enzyme linked to cell growth and proliferation/Akt cell survival pathway [6–9]. Two ligands, insulin-like growth factor (IGF)-1 and IGF-2, bind and activate the intrinsic tyrosine kinase activity of the IGF1R. Activated Akt phosphorylates a number of target proteins involved in a diverse spectrum of cellular activities, spanning cell-cycle progression, survival, metabolism, translation, and motility. In most cells, PI_3_K Akt signaling pathway is the dominant pathway in response to IGF stimulation, and many types of tumor cells depend on this pathway to be resistant to the apoptosis induced by chemotherapy, radiation, and antihormonal therapy.

Ganitumab, a fully human monoclonal IgG1 antibody against human IGF1R, exerts its antitumor activity by blocking ligand binding (IGF-1 and IGF-2) to induce receptor internalization and degradation without cross-reacting with the insulin receptor [10]. Blockade of IGF1R signaling by ganitumab inhibited activation of PI_3_K Akt pathway, leading to cell growth inhibition. In vivo studies demonstrated anti-cancer effect of ganitumab in human xenograft tumor models of sarcoma, breast carcinoma, colon carcinoma, ovarian carcinoma, and pancreatic carcinoma. Ganitumab also showed additive effects on the antitumor activity in combination with irinotecan, gemcitabine, cyclophosphamide, panitumumab, erlotinib, or gefitinib compared to each agent alone.

Based on these pre-clinical observations, ganitumab was applied to a clinical study to investigate safety and tolerability in patients with advanced solid tumors in the United States [11]. Here, we report the results of an open-label, multicenter, dose escalation phase 1 study to assess the safety, tolerability, pharmacokinetics (PK), anti-ganitumab antibodies, pharmacodynamics (PD), and antitumor activity of ganitumab in adult Japanese patients with relapsed and refractory solid tumors as investigated in the United States study.

## Materials and methods

### Eligibility criteria

This study was conducted in accordance with the ethical principles of the Declaration of Helsinki and was consistent with Good Clinical Practice and all applicable laws and regulations. Institutional Review Board approval and written informed consents were obtained before any study-specific procedures were started. Inclusion criteria included age of 20–74 years old; histologically or cytologically confirmed advanced solid tumors refractory to standard treatment or for which no standard therapy was available; Eastern Cooperative Oncology Group (ECOG) performance status ≤1; and adequate hematologic, hepatic, and renal functions. Exclusion criteria included hematological malignancies; central nervous system metastasis; uncontrolled diabetes; uncontrolled disease related to cardiac function (New York Health Association class > II); cardiac arrhythmia; and antitumor treatment within 4 weeks of study enrollment (or within 8 weeks for antibody therapy).

### Study design

This was an open-label, multicenter, dose escalation phase 1 study. Patients meeting the enrollment criteria received the administration of ganitumab treatments on Day 1, 15, and 29 followed by a 28-day treatment-free period (There was no treatment on Day 43). Patients received ganitumab every 2 weeks (Q2 W) starting on Day 57 until disease progression, unacceptable toxicities, or withdrawal of consent occurred. Patients were enrolled sequentially into 3 dose cohorts (6, 12, or 20 mg/kg; 6 patients per cohort). Ganitumab was administered by intravenous (IV) infusion over 60 min. The dose escalation to the next cohort was decided if none or 1 of the initial 6 patients experienced a dose-limiting toxicity (DLT) during the first 28 days of the study treatment. If 2 of 6 patients in a cohort experienced a DLT, 3 additional patients were added to the same cohort. If 2 of 9 patients in a cohort experienced a DLT, then dose escalation to the next specified cohort was to proceed as planned. If patients experienced grade 3 or 4 toxicities not considered related to the study treatment, ganitumab treatment was postponed until the toxicity was resolved to grade 1 or recovered to the patient’s baseline levels.

### Evaluation of safety

Adverse events were graded using National Cancer Institute Common Terminology Criteria for Adverse Events, version 3.0. In order to be considered a DLT, the toxicity must have occurred during the first 28 days after the initial administration of ganitumab. Any grade 4 or higher treatment-related hematologic toxicity (with the exception of leukopenia, neutropenia, and thrombocytopenia) was considered a DLT. To be considered a DLT, leukopenia and neutropenia must have been grade ≥4 for more than 3 days and thrombocytopenia must have been grade ≥4 lasting more than 7 days or grade ≥3 with grade ≥2 bleeding or grade ≥3 requiring transfusion of platelets. Any grade 3 or higher non-hematologic treatment-related toxicity was considered a DLT with the exception that grade 3 hyperglycemia and infusion reaction were not considered to be a DLT and that aspartate aminotransferase (AST) or alanine aminotransferase (ALT) had to be >10× institutional upper limit of normal to be considered a DLT.

### Pharmacokinetics

Blood samples were collected on Day 1 and 29 (predose, 0.5, 1 [within 10 min following infusion completion], 24, 48, 96, 168, 240, and 336 h postdose). In addition, blood samples were collected on Day 15 and 57 at predose, Day 50, every 4 weeks after Day 57, every 8 weeks after Day 113, and at 4 weeks following the last dose (as follow-up). On Day 15, a postdose blood sample was collected within 10 min following infusion completion. Ganitumab in serum was quantified using a validated double anti-idiotypic antibody sandwich immunoassay method [11]. PK parameters of ganitumab were estimated for each patient using noncompartmental methods with Phoenix WinNonlin Version 6.1 (Pharsight, Mountain View, CA).

### Anti-ganitumab antibodies assay

Blood samples were collected at predose on Day 1, 29, 57, 113, and every 8 weeks after Day 113. The following validated methods were used for anti-ganitumab antibody assay [11]. First, the presence of anti-ganitumab binding antibodies in serum was confirmed using a Meso Scale Discovery (MSD) electrochemiluminescence-based immunoassay (Meso Scale Discovery, Gaithersburg, MD). All serum samples positive for anti-ganitumab binding antibodies were additionally evaluated for potential neutralizing capabilities in the cell-based assay.

### Pharmacodynamics

PD samples were collected on Day 1 (predose), 2, 3, 5, 8, 11, 15 (predose), 29 (predose), 31, 33, 39, 43, 50, 106, and every 8 weeks after Day 106. IGF-1 and insulin-like growth factor binding protein-3 (IGFBP-3) were measured with a competitive binding radioimmunoassay. Growth hormone (GH) was measured 2-site immunometric procedure using monoclonal antibodies to 2 distinct epitopes of the hGH molecule.

### Evaluation of tumor response

Tumor response was evaluated by computed tomography or magnetic resonance imaging at Day 50, and every 8 weeks after Day 50 according to the Response Evaluation Criteria in Solid Tumors (RECIST) 1.0. Patients with a best response of stable disease needed to be confirmed after at least 6 weeks after initial administration.

## Results

### Patient characteristics

Twenty patients were enrolled between May 29, 2009 and October 12, 2010. One patient was withdrawn from the study before receiving any doses of ganitumab due to serious adverse events associated with disease progression. A total of 19 patients received ganitumab at a dose of 6 mg/kg (6 patients), 12 mg/kg (7 patients), or 20 mg/kg (6 patients). Patient characteristics are listed in Table 1. Median age was 58.0 years. All patients had good performance status and had received prior anti-cancer treatments specified in the protocol; 6 patients (32 %) received prior radiotherapy for cancer.

**Table 1:** Demographics and other baseline characteristics

	Ganitumab			
	6 mg/kg (*N* = 6)	12 mg/kg (*N* = 7)	20 mg/kg (*N* = 6)	All Subjects (*N* = 19)
Sex—*n* (%)				
Male	4 (67)	5 (71)	2 (33)	11 (58)
Female	2 (33)	2 (29)	4 (67)	8 (42)
Age—year				
Median	61.5	64.0	53.0	58.0
Range	50–68	43–70	28–62	28–70
ECOG performance status—*n* (%)				
0	5 (83)	6 (86)	3 (50)	14 (74)
1	1 (17)	1 (14)	3 (50)	5 (26)
Prior anti-cancer radiotherapy—*n* (%)				
Yes	3 (50)	2 (29)	1 (17)	6 (32)
No	3 (50)	5 (71)	5 (83)	13 (68)
Primary tumor type—*n* (%)				
Neoplasm, breast	1 (17)	1 (14)	2 (33)	4 (21)
Neoplasm, stomach	0 (0)	2 (29)	1 (17)	3 (16)
Carcinoma, non-small cell lung (NSCLC)	2 (33)	0 (0)	0 (0)	2 (11)
Neoplasm, rectal	1 (17)	0 (0)	1 (17)	2 (11)
Thymic carcinoma	0 (0)	2 (29)	0 (0)	2 (11)
Neoplasm, colon	0 (0)	1 (14)	0 (0)	1 (5)
Neoplasm, esophageal	1 (17)	0 (0)	0 (0)	1 (5)
Neoplasm, kidney	1 (17)	0 (0)	0 (0)	1 (5)
Neoplasm, small intestine	0 (0)	1 (14)	0 (0)	1 (5)
Neoplasm, uterine	0 (0)	0 (0)	1 (17)	1 (5)
Sarcoma, soft tissue	0 (0)	0 (0)	1 (17)	1 (5)

### Safety

Ganitumab was well tolerated at all doses evaluated. No DLTs were observed and the maximum tolerated dose was not identified. Eighteen patients (95 %) had ≥1 adverse event during the all treatment course of this study. Across all cohorts, the most common treatment-emergent adverse events (occurring in >20 % of patients) were fatigue, infusion-related reaction and neutropenia (8 patients; 42 %, each), leukopenia (7 patients; 37 %), thrombocytopenia (6 patients; 32 %), vomiting (5 patients; 26 %), AST increased and nausea (4 patients; 21 %, each) (Table 2), and all of them were mostly mild or moderate in severity. Grade 3 adverse events were observed in 6 patients (32 %). Grade 4 adverse events were observed in 2 patients (11 %); neutropenia in the 12 mg/kg cohort and hyponatremia in the 6 mg/kg cohort (1 patient; 5 %, each). Three patients (16 %) had serious adverse events: dyspnoea (1 patient in the 6 mg/kg cohort, grade 3), respiratory tract hemorrhage (1 patient in the 12 mg/kg cohort; grade 2), and pleural effusion (1 patient in the 20 mg/kg cohort; grade 3). Only respiratory tract hemorrhage in a patient with thymic carcinoma was considered to be related to ganitumab by the investigator. Eighteen patients (95 %) had treatment-related adverse events. The most common treatment-related adverse events (occurring in >15 % of patients) were infusion-related reaction and neutropenia (8 patients; 42 %, each), leukopenia (7 patients; 37 %), thrombocytopenia (6 patients; 32 %), AST increased, fatigue, nausea, and vomiting (4 patients; 21 %, each), ALT increased, lymphopenia, rash, and stomatitis (3 patients; 16 %, each). Most treatment-related adverse events were grade 1 (4 patients; 21 %), grade 2 (8 patients; 42 %), or grade 3 (5 patients; 26 %) at worst severity. One patient (5 %) had a grade 4 treatment-related adverse event (neutropenia in the 12 mg/kg cohort). There were no fatal adverse events. No patients had adverse events causing discontinuation of ganitumab. Reasons for ganitumab discontinuation were disease progression (17 patients; 85 %), physician decision (2 patients; 10 %). Infusion-related reaction was reported in 8 patients (42 %). All infusion reactions were grade 1 or 2 in severity and non-serious, and no patient withdrew from the study because of infusion-related reaction. Eight patients (42 %) had neutropenia events (neutropenia and leukopenia). Four patients had grade 3 or grade 4 neutropenia. Three patients had grade 3 leukopenia. There were no serious neutropenia events. No clinically significant trends in serum chemistry or hematology laboratory values were observed, with the exception of neutrophils, leukocytes, and platelet counts. No clinically significant trends in QT/QTc prolongation were observed.

**Table 2:** Treatment-emergent adverse events occurring in ≥3 subjects

	Ganitumab			
Preferred term	6 mg/kg (*N* = 6) *n* (%)	12 mg/kg (*N* = 7) *n* (%)	20 mg/kg (*N* = 6) *n* (%)	All Subjects (*N* = 19) *n* (%)
Subjects with any treatment-emergent adverse events	6 (100)	6 (86)	6 (100)	18 (95)
Fatigue	4 (67)	3 (43)	1 (17)	8 (42)
Infusion-related reaction	2 (33)	4 (57)	2 (33)	8 (42)
Neutropenia	4 (67)	1 (14)	3 (50)	8 (42)
Leukopenia	4 (67)	1 (14)	2 (33)	7 (37)
Thrombocytopenia	0 (0)	2 (29)	4 (67)	6 (32)
Vomiting	1 (17)	3 (43)	1 (17)	5 (26)
Aspartate aminotransferase increased	0 (0)	3 (43)	1 (17)	4 (21)
Nausea	0 (0)	2 (29)	2 (33)	4 (21)
Alanine aminotransferase increased	0 (0)	2 (29)	1 (17)	3 (16)
Anorexia	0 (0)	1 (14)	2 (33)	3 (16)
Back pain	1 (17)	1 (14)	1 (17)	3 (16)
Headache	1 (17)	0 (0)	2 (33)	3 (16)
Lymphopenia	0 (0)	1 (14)	2 (33)	3 (16)
Pyrexia	2 (33)	1 (14)	0 (0)	3 (16)
Rash	0 (0)	3 (43)	0 (0)	3 (16)
Stomatitis	2 (33)	0 (0)	1 (17)	3 (16)

Three patients (16 %) tested positive for binding antibodies at baseline of whom one also tested positive in a post-treatment binding antibody test. Therefore, no patient developed anti-ganitumab binding antibodies during this study. A bioassay was performed in all 3 patients who tested positive for binding antibodies. No neutralizing antibodies were detected in any of the binding antibody positive patients based on this cell-based bioassay.

### Pharmacokinetics

**Fig. 1:**
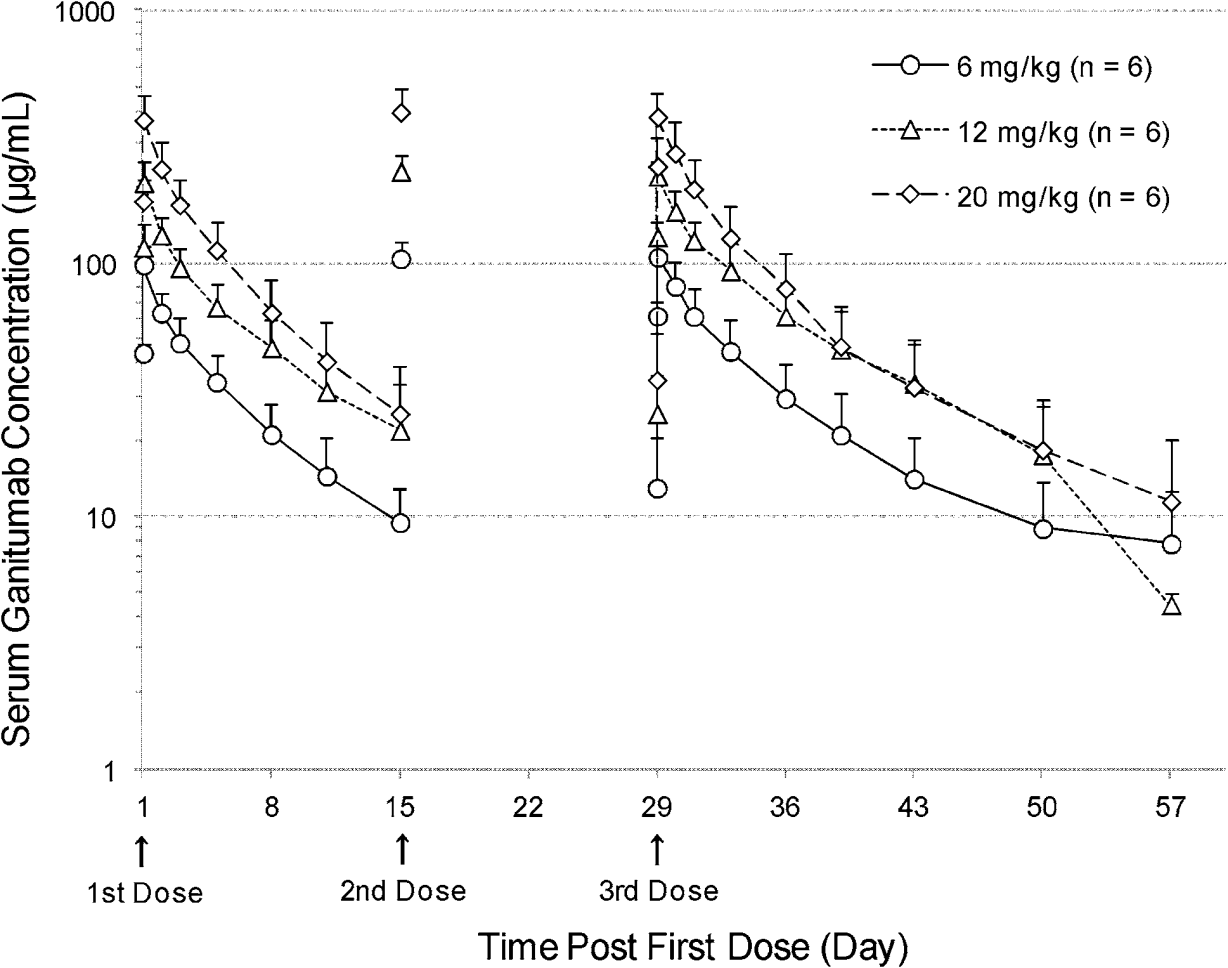
Mean (+SD) Serum ganitumab concentration–time profiles after biweekly intravenous administrations of ganitumab. Pharmacokinetic samples were collected on Day 1 and 29 at predose and postdose at 0.5, 1, 24, 48, 96, 168, 240, and 336 h. In addition, PK samples were also collected on Day 15 and 57 at predose, and Day 50

The serum concentration–time profiles and the PK parameters of ganitumab are shown in Fig. 1 and Table 3, respectively. After administrations of ganitumab at 6, 12, and 20 mg/kg, the maximal serum concentration of ganitumab was observed at the infusion completion (approximately 1 h postdose) and then declined with mean terminal elimination half-life (*t*_1/2_) of 6.4–9.1 days in Cycle 3. The mean maximum observed concentration (*C*_max_) and area under the serum concentration–time curve (AUC) appear to increase with the dose-proportion manner at the dose range. In Cycle 3, the mean volume of distribution at steady state (*V*_ss_) was 92–120 mL/kg, and the mean systemic clearance (CL) was 12–14 mL/day/kg, at the 3 dose levels. The accumulation ratio of AUC was estimated at 1.1–1.3, when the AUC in Cycle 3 was compared to that in Cycle 1.

**Table 3:** Noncompartmental pharmacokinetic parameters of ganitumab

Dose	Cycle 1				Cycle 3							
	*T*_max_ (h)	*C*_max_ (μg/mL)	*C*_336_ (μg/mL)	AUCt (day·μg/mL)	*T*_max_ (h)	*C*_max_ (μg/mL)	*C*_336_ (μg/mL)	AUCt (day·μg/mL)	*t*_1/2_ (day)	*V*_ss_ (mL/kg)	CL (mL/day/kg)	AR
6 mg/kg	*n* = 6				*n* = 6							
Mean	1.167*	98.72	9.468	402.4	1.083*	106.0	14.07	525.5	9.098	120.0	12.40	1.297
SD	1.017, 1.550*	16.80	3.365	99.15	1.033, 1.133*	16.24	6.543	155.8	2.076	38.84	4.161	0.1080
12 mg/kg	*n* = 6				*n* = 6							
Mean	1.092*	208.7	21.90	836.4	1.067*	221.3	33.55	1,102	8.109	109.0	11.54	1.315
SD	0.9833, 1.200*	45.12	11.32	178.8	1.017, 1.250*	27.93	14.77	272.8	1.941	29.26	3.211	0.1270
20 mg/kg	*n* = 6				*n* = 5							
Mean	1.117*	368.8	25.44	1,334	1.050*	380.2	32.46	1,522	6.440	92.31	14.48	1.148
SD	1.067, 1.383*	95.75	14.02	364.7	0.7833, 1.167*	88.10	17.75	508.5	2.013	15.99	5.196	0.07959

* *T*_max_ reported as median and range. *T*_max_ time to reach *C*_max_, *C*_max_ maximum observed concentration after IV infusion, *C*_336_ observed concentration at 336 h postdose

*AUCt* area under the concentration–time curve for a dosing interval (336 h with Q2 W regimen), *t*_1/2_ terminal elimination half-life

*V*_ss_ volume of distribution at steady state, *CL* systemic clearance, *AR* accumulation ratio of AUC, *AUC* AUCt _Day29_/AUCt _Day 1_

### Pharmacodynamics

**Fig. 2:**
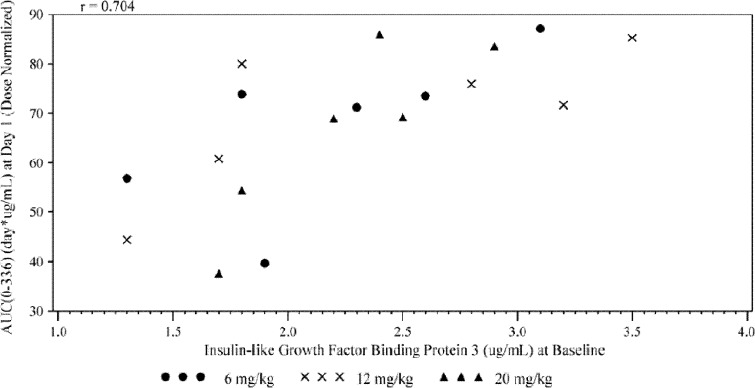
Scatter plot for IGFBP-3 at baseline and AUC(0-336) at first dosing

Serum IGF-1 and IGFBP-3 increased transiently after ganitumab administration in most of the patients tested but not dose proportionally. On the other hand, serum GH did not change in some patients, and no clear results were obtained. We investigated whether these PD markers could be prognostic or predictive factors; focused on the baseline levels of total IGF-1 and IGFBP-3 and maximum proportional changes from the baseline; and analyzed them across a number of parameters. We plotted baseline levels of IGF-1 or IGFBP-3, and maximum percent tumor reduction, time to progression, maximum proportional changes in neutrophil or platelet counts, AUC of ganitumab, minimum observed concentration, *C*_max_, or *t*_1/2_, *z* (data not shown). Correlation was found between baseline of IGFBP-3 and ganitumab AUC at first dosing, *r* = 0.704 (Fig 2), while no clear correlation was found in other factors tested.

### Efficacy

The tumor response was assessed in 19 patients. All patients had baseline measurable disease. No patients showed a best response of complete response or partial response. Seven patients (37 %) had a best response of stable disease, 3 patients in the 6 mg/kg cohort, 2 patients in the 12 mg/cohort, and 2 patients in the 20 mg/kg cohort. Disease control rate (complete response + partial response + stable disease) was 37 % (95 % confidence interval, 16.3–61.6). The tumor type which the patient with stable disease had was thymic carcinoma, non-small cell lung cancer, breast cancer, soft tissue sarcoma, and stomach cancer.

## Discussion

This was the phase 1 clinical study of ganitumab to evaluate the safety and PK in Japanese patients with advanced tumors. Ganitumab up to 20 mg/kg was tolerable in Japanese patients with advanced solid tumors. Three patients had serious adverse events: dyspnoea, respiratory tract hemorrhage, and pleural effusion; however, all the events were grade 2 or 3. Among them, respiratory tract hemorrhage was thought due to the primary disease; however, grade 2 thrombocytopenia also occurred after administration of ganitumab; therefore, the investigator determined that there is a relationship with ganitumab.

Based on the data from other studies with ganitumab, a number of adverse events have been identified including hyperglycemia, thrombocytopenia, hepatotoxicity, rash, and infusion reactions. Most of these adverse events were well tolerated and resolved during the study without any medication. Infusion reactions were reported in 8 patients. The event of infusion reactions includes infusion-related reaction manifested by chill, shivering, dyspnoea, headache, and fever (8 patients) during the all treatment courses, and vomiting (2 patients), blood pressure decreased, hypertension, and nausea (1 patient, each) with the first administration of ganitumab. All of these events were grade 1 or 2 and non-serious. Thrombocytopenia was reported in 6 patients and was more likely to occur with higher doses, suggesting the dose-dependent manner. While neutropenia was reported in 8 patients, dose dependence with neutropenia was not observed. Neutropenia had a higher incidence compared with the results of the phase 1, for the non-Japanese, first in human study [11]. Although there was neutropenia of grade 3 or 4 in 4 patients, they recovered before the next administration (2 weeks later); therefore, it is considered to be manageable. IGF-1 was reported to delay the neutrophil apoptosis through PI_3_K pathway [12]. This finding could be partially explained neutropenia induced by ganitumab.

The exposure of ganitumab appears to increase in a dose-proportion manner in this study, following IV administrations at doses of 6, 12, and 20 mg/kg. No remarkable difference in the mean values of CL and *V*_ss_ was observed among the 3 doses. These findings indicate that ganitumab exhibited almost linear PK at the dose range, 6–20 mg/kg. The accumulation ratio of ganitumab AUC was approximately one, suggesting that the Q2 W regimen of ganitumab would result in negligible accumulation and achieve nearly steady state after a few doses. The mean values of PK parameters of ganitumab in this study were nearly close to those in the overseas first in human study; the mean CL was 8.6–15 mL/day/kg and the accumulation ratio of AUC was 1.1–1.4 in Cycle 3 following IV administrations Q2 W at doses of 1, 3, 10, 12, and 20 mg/kg [11]. Therefore, the PK profile of ganitumab in Japanese cancer patients is considered to be comparable to non-Japanese cancer patients at the dose range.

Serum IGF-1 and IGFBP-3 increased after ganitumab administration in most of the patients tested as observed in other IGF1R targeted therapy [8]. However, these increases were not dose dependent.

Most of IGF-1 in the blood is bound to IGFBPs and the major IGFBP is IGFBP-3 [13, 14]. IGFBP-3 binds to IGF-1 and modulates its activity [15, 16]. Blockade of IGF1R resulted in a compensatory increase in the levels of circulating IGF-1 and IGFBP-3. Baseline levels of these and other components of the IGF axis may also reflect the activation status of the IGF1R pathway. In addition, the effect of ganitumab might be associated with exposure; therefore, we examined the relationship between AUC and circulating markers. Baseline IGFBP-3 levels correlated with the AUC of ganitumab but no other clear correlations were observed. High levels of baseline IGFBP-3 were associated with favorable overall survival in ganitumab-treated patients and may predict a treatment effect with metastatic pancreatic cancer [17]. The relationship between circulating PD markers, AUC, and efficacy of ganitumab should be investigated in the future studies.

This was a phase 1 study, and tumor response was not a primary endpoint. No patients had a best response of complete response or partial response, although a patient with thymic carcinoma received 6 mg/kg of ganitumab until progression shortly after week 47.

Clinical development of ganitumab is advancing for pancreatic cancer [10]. In Japan, the phase 1b study, the tolerability of combination therapy with gemcitabine and ganitumab 20 mg/kg for metastatic pancreatic cancer was confirmed [18]. In addition, the combination of gemcitabine and ganitumab 12 mg/kg resulted in trends toward longer progression-free survival and longer overall survival for metastatic pancreatic cancer in the non-Japanese phase 2 study [19]. Based on these results, global phase 3 study is being conducted for metastatic pancreatic cancer.

In summary, ganitumab showed acceptable safety as a monotherapy at IV doses up to 20 mg/kg Q2 W in Japanese patients with advanced solid tumors. Clinical evaluations of ganitumab in combination with either molecularly targeted agents or chemotherapy are in progress.
